# Role of the Insulin Receptor in Mediating Cytosolic Delivery of Proteins by a Modified Cell-Penetrating Peptide

**DOI:** 10.3390/ph18121885

**Published:** 2025-12-12

**Authors:** Keito Sugai, Akiko Okuda

**Affiliations:** Graduate School of Health Sciences, Niigata University, 2-746 Asahimachi-dori, Chuo-ku, Niigata 951-8518, Japan

**Keywords:** cell-penetrating peptide, insulin receptor, insulin-like growth factor 1 receptor, extracellular signal-regulated kinase 1/2, endocytosis, enhanced green fluorescent protein

## Abstract

**Background:** Intracellular delivery of high-molecular-weight proteins is limited by the cell membrane. Cell-penetrating peptides (CPPs) offer a potential solution, but effective cytosolic delivery remains hindered by endosomal sequestration. Pas2r12, a CPP-derived peptide, facilitates cytosolic delivery of proteins including immunoglobulin G. Because Pas2r12 internalization occurs via caveolae-dependent endocytosis, we hypothesized that cell-surface receptors contribute to uptake. **Methods:** HEK293 cells were treated with Pas2r12 alone or complexed with enhanced green fluorescent protein (EGFP). Phosphorylation of insulin receptor (INSR), insulin-like growth factor 1 receptor (IGF1R), and extracellular signal–regulated kinase 1/2 (ERK1/2) was analyzed by Western blot. Linsitinib was used to inhibit INSR/IGF1R kinase activity. Cytosolic delivery was assessed by confocal microscopy, and receptor involvement was evaluated using siRNA-mediated knockdown and receptor overexpression. **Results:** Pas2r12 alone transiently increased INSR/IGF1R phosphorylation at 2 min (6.6-fold), which was suppressed by linsitinib (1.3-fold), and strongly increased ERK1/2 phosphorylation (6.2-fold), which was not inhibited by linsitinib. Pas2r12–EGFP did not induce detectable INSR/IGF1R phosphorylation in parental cells but increased ERK1/2 phosphorylation (3.4-fold). Linsitinib markedly reduced cytosolic EGFP delivery to 16% of control. INSR knockdown decreased delivery to 13–16%, and IGF1R knockdown to 19–65%. In INSR-overexpressing lines, Pas2r12–EGFP induced INSR/IGF1R phosphorylation (6.0-fold) and enhanced delivery (230–270%). In IGF1R-overexpressing lines, Pas2r12–EGFP did not induce phosphorylation, and delivery decreased to 60–69%. **Conclusions:** Pas2r12-mediated cytosolic delivery involves both INSR and IGF1R, with INSR contributing more prominently. These findings, including the largely INSR/IGF1R-independent ERK1/2 activation, provide mechanistic insight into Pas2r12-mediated protein delivery.

## 1. Introduction

The intracellular delivery of high-molecular-weight proteins is used to analyze cell function and advance drug discovery. However, because the cell membrane presents a barrier to the permeation of macromolecules, developing safe and efficient delivery methods is challenging. Cell-penetrating peptides (CPPs) have emerged as a promising approach to address this limitation [1,2]. CPPs can cross cellular membranes and facilitate the intracellular delivery of various cargos, including proteins, nucleic acids, and small molecules [3]. Despite these advantages, improving the specificity and efficiency of CPP-mediated delivery has been challenging.

A primary limitation of CPP-mediated delivery is that most cargos are internalized through endocytosis [4]. Although endocytosis enables initial entry, the subsequent release of cargo from the endosomes into the cytosol is frequently inefficient. Therefore, endosomal entrapment and degradation represent major obstacles that restrict macromolecule delivery and limit the effectiveness of CPP-based intracellular delivery strategies. To address these challenges, numerous strategies have been used, including incorporating pH-responsive endosomal escape motifs, such as HA2, GALA, and histidine-rich sequences; introducing chemical modifications that enhance membrane disruption or promote proton sponge effects; and engineering CPP structures that favor direct translocation or reduce endocytic uptake [5,6,7]. However, despite these advances, the upstream mechanisms governing CPP-mediated signaling and endocytic entry are poorly defined, particularly with respect to receptor-mediated processes.

We had identified an optimized CPP, Pas2r12, which complexes with enhanced green fluorescent protein (EGFP; 27 kDa) or immunoglobulin G (IgG; 150 kDa) to facilitate their intracellular delivery [8]. Pas2r12 was designed based on the Pas motif (FFLIPKG) [9] and PasΔPK [10]. It contains two tandem FFLIG sequences (FFLIGFFLIG), followed by a stretch of 12 d-arginine residues, yielding a full peptide sequence of FFLIGFFLIGrrrrrrrrrrrr. Pas2r12 mediates the cytosolic delivery of cargo proteins through caveolae-dependent endocytosis. Caveolae are flask-shaped invaginations of the plasma membrane, approximately 80 nm in diameter, that contribute to cellular uptake, stress responses, and signal transduction [11,12,13]. This pathway is typically triggered by specific interactions between ligands and cell surface receptors, as seen when Simian virus 40 binds to GM1 ganglioside [14] and albumin to gp60 [15]. However, the molecular basis of Pas2r12-mediated intracellular delivery, including its cell surface receptor(s), is unclear.

Phosphoproteomic analyses have revealed that Pas2r12 treatment induces both early and sustained phosphorylation of extracellular signal–regulated kinase 1/2 (ERK1/2)—a key component of the mitogen-activated protein kinase (MAPK) pathway [16]. Phosphorylation occurred following stimulation with Pas2r12 and with Pas2r12–EGFP complexes, indicating that Pas2r12–EGFP activates intracellular signaling cascades, particularly through the MAPK pathway. These findings indicate that ERK1/2 plays a central role in the cellular response triggered by Pas2r12–EGFP. ERK1/2 is a representative MAPK that regulates cell proliferation, differentiation, and stress responses; its sustained activation by Pas2r12–EGFP may reflect a signaling event associated with protein delivery and receptor stimulation.

In this study, we aimed to clarify the receptor(s) that mediate Pas2r12–EGFP–induced ERK1/2 activation and caveolae-dependent endocytosis. Identifying the receptor(s) is essential for understanding the basis of Pas2r12-mediated protein delivery. We hypothesize that Pas2r12–EGFP interacts with one or more membrane receptors that function upstream of ERK1/2 activation and caveolae-dependent endocytosis. Because insulin receptor (INSR) and insulin-like growth factor 1 receptor (IGF1R) regulate both ERK1/2 signaling and caveolae-dependent endocytosis, they represent receptor candidates for Pas2r12–EGFP.

Among receptor tyrosine kinases, INSR and IGF1R are strong candidates for involvement in Pas2r12–EGFP uptake because both are upstream activators of MAPK/ERK signaling [17,18]. Both INSR and IGF1R participate in caveolae-dependent endocytosis [19,20] and are among the few receptor tyrosine kinases that regulate both ERK1/2 signaling and caveolae-mediated internalization, making them potential receptor candidates for Pas2r12–EGFP.

INSR and IGF1R are heterotetrameric receptors that comprise two extracellular α-subunits and two transmembrane β-subunits [21,22]. The α-subunits mediate ligand binding at the cell surface; the β-subunits contain intracellular kinase domains that undergo autophosphorylation upon ligand engagement, initiating downstream signaling cascades. INSR and IGF1R preferentially bind insulin and IGF-1, respectively; however, ligand cross-reactivity can occur depending on the cellular context [23,24]. Both receptors localize to caveolae-rich membrane domains, positioning them to coordinate signal transduction with membrane trafficking events [19,20].

Because INSR and IGF1R regulate both ERK1/2 signaling and caveolae-dependent endocytosis, they represent receptor candidates for Pas2r12–EGFP. Although phosphoproteomic analyses did not detect phosphorylation of INSR or IGF1R following Pas2r12–EGFP stimulation [16], this absence may reflect transient or low-abundance phosphorylation events that were undetected under other experimental conditions. Therefore, we employed modified detection conditions and performed Western blot to capture transient or low-abundance phosphorylation events missed earlier.

To evaluate the potential roles of INSR and IGF1R in Pas2r12-mediated intracellular delivery, we performed pharmacological inhibition using linsitinib [25], knockdown, and receptor overexpression. The results indicate that the expression and activation of INSR contribute substantially to delivery efficiency. These findings provide insights into the receptor-associated mechanisms of novel Pas2r12-mediated protein transport and may support the refinement of protein delivery technologies.

## 2. Results

### 2.1. Phosphorylation of INSR/IGF1R Following Pas2r12 Stimulation

INSR and IGF1R share ~70% amino acid sequence homology [26] and contain conserved autophosphorylation sites in their tyrosine kinase domains—Y1162/Y1163 in INSR [27] and Y1135/Y1136 in IGF1R [28]. To determine whether Pas2r12 induces the activation of these receptors, we examined phosphorylation at these specific sites following Pas2r12 stimulation.

**Figure 1:**
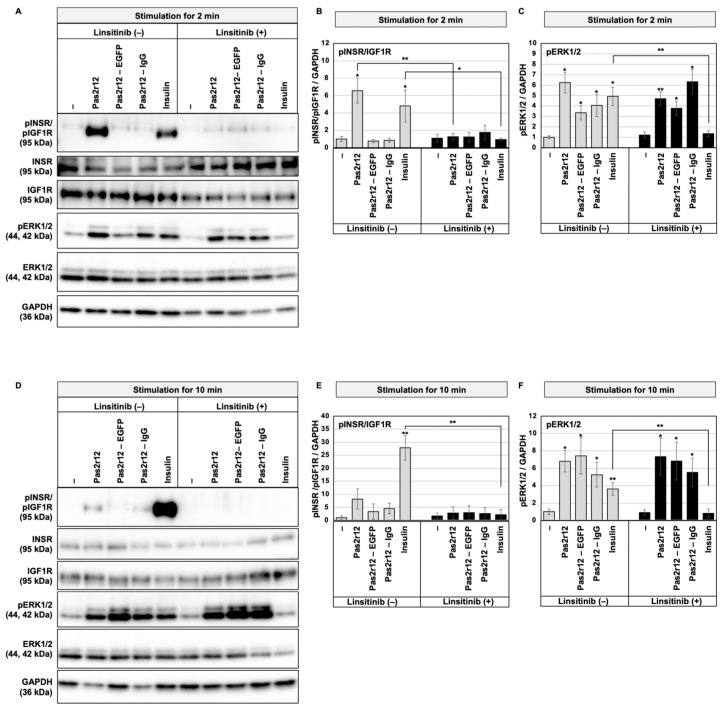
Effects of Pas2r12 or Pas2r12–cargo protein complex-mediated stimulation on INSR/IGF1R and ERK1/2. HEK293 cells were pretreated with dimethyl sulfoxide (linsitinib [−]) or with linsitinib (linsitinib [+]) and subsequently stimulated with Pas2r12, Pas2r12–EGFP, Pas2r12–IgG, or insulin for 2 min (**A**–**C**) or 10 min (**D**–**F**). Panels A and D show representative Western blot images. Panels B and E display the phosphorylation levels of INSR/IGF1R (pINSR/pIGF1R); panels C and F show the phosphorylation levels of ERK1/2 (pERK1/2); all values were normalized to GAPDH. Phosphorylation levels were analyzed and compared with the solvent control (DMEM only, linsitinib [−]) using Student’s *t*-test. For each treatment condition, linsitinib (+) was compared with the corresponding linsitinib (−) condition. Statistically significant differences compared with the control are indicated on the bar graphs; significant differences between linsitinib (+) and linsitinib (−) treatments are marked by horizontal lines. Statistical comparisons were performed against control cells using Student’s *t*-test. Error bars indicate the standard error of the mean (SEM). * *p* < 0.05 and ** *p* < 0.01. *N* = 4.

HEK293 cells were stimulated for 2 min with Pas2r12, Pas2r12–EGFP, or Pas2r12–IgG. Phosphorylation levels were analyzed by Western blot using phosphospecific antibodies against INSR Y1162/Y1163 and IGF1R Y1135/Y1136 and compared with a vehicle control containing 0.01% dimethyl sulfoxide (DMSO). Stimulation with Pas2r12 significantly increased INSR/IGF1R phosphorylation (6.6-fold; Figure 1A,B); this effect was significantly suppressed by linsitinib (1.3-fold). Under the same conditions, Pas2r12–EGFP and Pas2r12–IgG did not significantly alter phosphorylation levels. As a positive control, 2-min insulin stimulation induced a significant increase in INSR/IGF1R phosphorylation (4.8-fold), which was inhibited by linsitinib (0.9-fold).

We examined the phosphorylation of ERK1/2—a downstream signaling molecule. Consistent with previous observations [16], 2-min stimulation with Pas2r12 or its complexes significantly increased ERK1/2 phosphorylation compared with the control (Pas2r12: 6.2-fold; Pas2r12–EGFP: 3.4-fold; and Pas2r12–IgG: 4.1-fold; Figure 1A,C). However, these responses were largely unaffected by linsitinib (Pas2r12: 4.7-fold; Pas2r12–EGFP: 3.8-fold; and Pas2r12–IgG: 6.3-fold). By comparison, insulin-induced ERK1/2 phosphorylation (4.9-fold) was suppressed by linsitinib (1.3-fold).

When the stimulation period was extended to 10 min, Pas2r12 no longer induced a detectable increase in INSR/IGF1R phosphorylation compared with the control (Figure 1D,E); insulin continued to produce strong phosphorylation (28-fold), which was inhibited by linsitinib (2.2-fold). By contrast, Pas2r12–EGFP and Pas2r12–IgG did not significantly affect phosphorylation levels.

Conversely, ERK1/2 phosphorylation was significantly elevated after 10 min of stimulation with Pas2r12 or its complexes (Pas2r12: 6.8-fold; Pas2r12–EGFP: 7.4-fold; and Pas2r12–IgG: 5.3-fold; Figure 1D,F). These effects were insensitive to linsitinib (Pas2r12: 7.3-fold; Pas2r12–EGFP: 6.8-fold; and Pas2r12–IgG: 5.5-fold). By contrast, insulin significantly activated both INSR/IGF1R and ERK1/2 at 10 min (3.6-fold); this effect was suppressed by linsitinib (0.8-fold). Collectively, Pas2r12 induced only a transient increase in INSR/IGF1R phosphorylation at early time points while producing sustained ERK1/2 activation that was largely insensitive to linsitinib; insulin activated both INSR/IGF1R and ERK1/2 in a linsitinib-sensitive manner. Stimulation with Pas2r12–EGFP and Pas2r12–IgG did not activate INSR/IGF1R; both complexes continued to induce robust ERK1/2 phosphorylation, indicating that Pas2r12–EGFP and Pas2r12–IgG can activate ERK1/2 signaling independent of detectable INSR/IGF1R activation.

### 2.2. Inhibition of Pas2r12-Mediated Cytosolic Delivery of EGFP by Linsitinib

**Figure 2:**
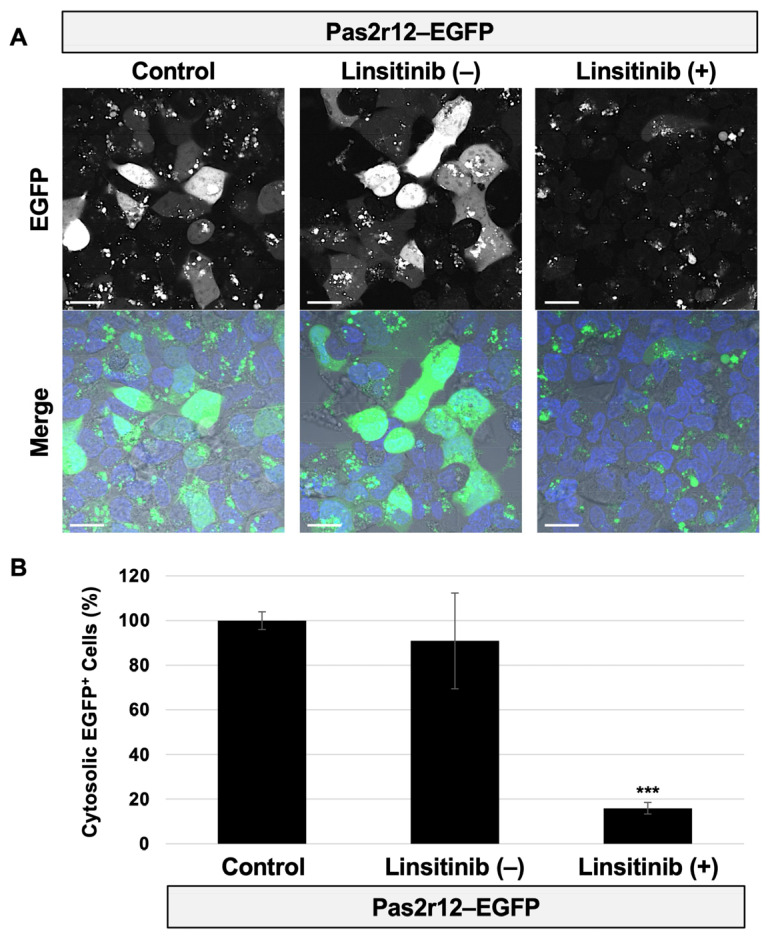
Effect of linsitinib on the Pas2r12-mediated cytosolic delivery of EGFP. HEK293 cells were pretreated with 0.01% DMSO (linsitinib [−]) or linsitinib (linsitinib [+]) and then incubated with the Pas2r12–EGFP complex before imaging. Untreated cells (DMEM without DMSO or linsitinib) were used as control. (**A**) Representative confocal laser scanning microscopy images. Merged images show EGFP fluorescence (green), nuclear staining (blue), and differential interference contrast. Scale bars represent 20 μm. (**B**) Percentage of cells exhibiting cytosolic EGFP delivery. Statistical comparisons were performed against control cells using Student’s *t*-test. Error bars indicate the standard error of the mean (SEM). *** *p* < 0.001. *N* = 4.

To determine whether signaling through INSR/IGF1R contributes to the Pas2r12-mediated cytosolic delivery of EGFP, cells were pretreated with linsitinib prior to incubation with the Pas2r12–EGFP complex. First, the cytosolic delivery efficiencies for EGFP and Pas2r12–EGFP were examined. EGFP was not delivered to the cytosol (0%); Pas2r12 induced cytosolic dispersion of EGFP in 26% of the cells (Figure S1). For all experiments, images were acquired under microscope settings that minimize background interference, including cellular autofluorescence. Delivery efficiency was quantified under these conditions. Compared to untreated cells (control, 100%), delivery efficiency was comparable in the vehicle control (91%, linsitinib [−]) but markedly reduced after linsitinib treatment (16%, linsitinib [+]) (Figure 2A,B). Although Pas2r12–EGFP did not produce detectable INSR/IGF1R phosphorylation, inhibition of INSR/IGF1R kinase activity significantly reduced the efficiency of cytosolic delivery.

### 2.3. Suppression of Pas2r12-Mediated Cytosolic Delivery by the Knockdown of INSR or IGF1R

**Figure 3:**
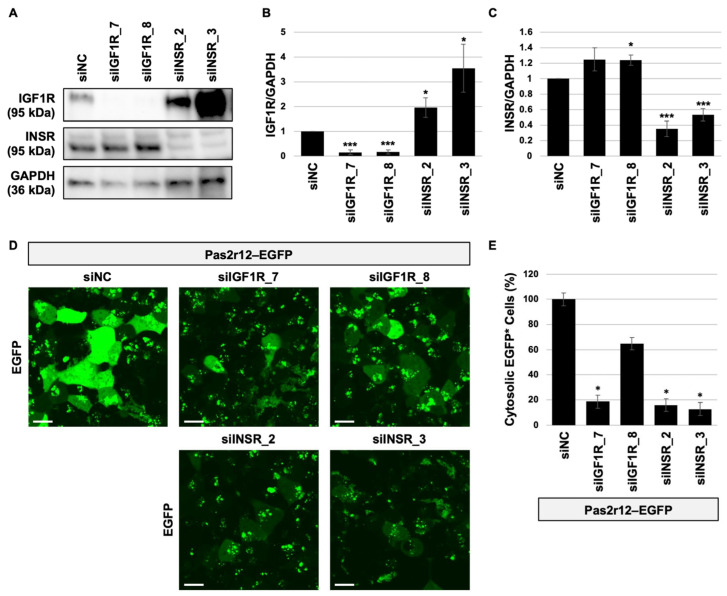
Effect of *IGF1R* and *INSR* knockdown on the Pas2r12-mediated cytosolic delivery of EGFP. Western blot analyses (**A**–**C**) and confocal laser scanning microscopy images (**D**,**E**). Graphs B and C show IGF1R and INSR expression levels, respectively, normalized to GAPDH. Cells analyzed with confocal laser scanning microscopy in panel A were analyzed by Western blot (**A**–**C**). (**D**) Pas2r12-mediated cytosolic delivery of EGFP in knockdown cells, with EGFP fluorescence shown in green, and (**E**) percentage of cells exhibiting cytosolic EGFP delivery. Scale bars represent 20 μm. Statistical comparisons were performed against siNC cells using Student’s *t*-test. Error bars indicate the standard error of the mean (SEM). * *p* < 0.05, *** *p* < 0.001. *N* = 4.

To evaluate the involvement of INSR and IGF1R in the Pas2r12-mediated cytosolic delivery of EGFP, targeted gene knockdown was performed using small interfering RNAs (siRNAs). *IGF1R* expression was targeted with siIGF1R_7 and siIGF1R_8; *INSR* expression was targeted with siINSR_2 and siINSR_3. Compared to the negative control siRNA (siNC), *IGF1R* levels were significantly reduced to 0.1- and 0.2-fold in cells transfected with siIGF1R_7 and siIGF1R_8, respectively (Figure 3A,B). Treatment with siIGF1R_7 did not alter INSR expression, and treatment with siIGF1R_8 modestly increased INSR levels (~1.2-fold, *p* = 0.04) (Figure 3A,C). Treatment with siINSR_2 and siINSR_3 significantly reduced INSR levels to 0.3- and 0.5-fold, respectively (Figure 3A,C). By contrast, treatment with siINSR_2 and siINSR_3 modestly increased IGF1R levels (~2.0- and 3.5-fold, respectively) (Figure 3A,B). These results demonstrate that knockdown of one receptor leads to the compensatory upregulation of the other, with the effect being more pronounced following INSR knockdown.

Pas2r12–EGFP complexes were added to knockdown cells to evaluate the efficiency of cytosolic delivery. Using siNC-treated cells as the reference (100%), delivery efficiency was reduced to 19% (siIGF1R_7), 65% (siIGF1R_8), 16% (siINSR_2), and 13% (siINSR_3) (Figure 3D,E). Excluding siIGF1R_8, knockdown of either receptor significantly reduced delivery efficiency, indicating that both INSR and IGF1R contribute functionally to Pas2r12-mediated cargo delivery.

### 2.4. Reduced Pas2r12-Mediated EGFP Delivery in IGF1R Overexpressing Cell Lines

**Figure 4:**
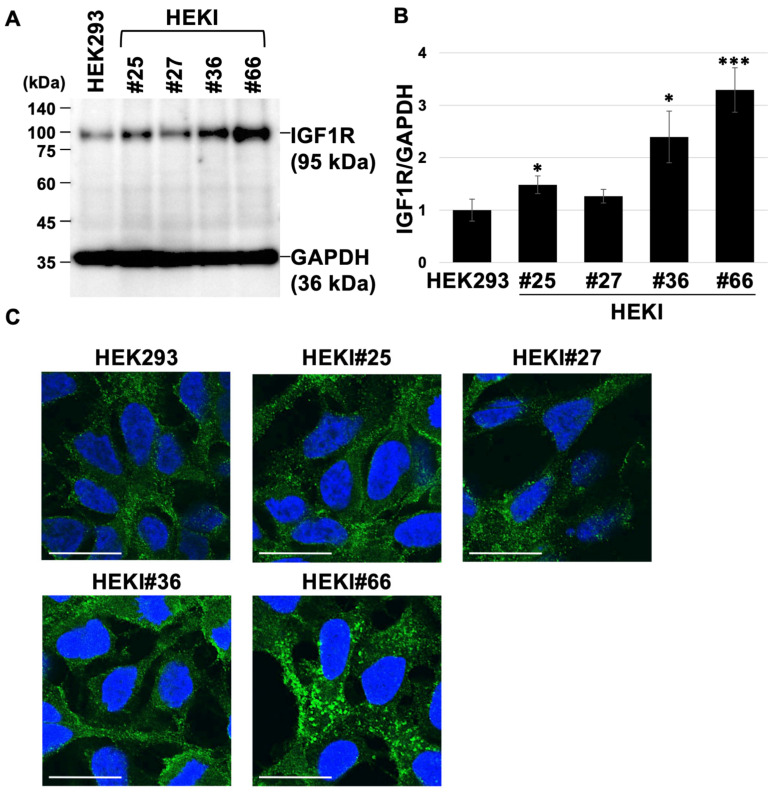
Assessment of IGF1R overexpression. (**A**) Verification of IGF1R expression levels in HEKI cells. (**B**) Relative levels of IGF1R expression normalized to GAPDH based on the data in panel A. Values for IGF1R/GAPDH are expressed compared with parental HEK293 cells (set to 1). Statistical comparisons were performed against HEK293 cells using Student’s *t*-test. Error bars indicate the standard error of the mean (SEM). * *p* < 0.05, *** *p* < 0.001. *N* = 3. (**C**) Subcellular localization of IGF1R is shown in green; nuclei were counterstained with Hoechst 33342 (blue). Scale bars represent 20 μm.

To evaluate the effect of IGF1R overexpression on the Pas2r12-mediated cytosolic delivery of EGFP, IGF1R levels were compared across engineered HEK293-derived overexpression lines. IGF1R expression was normalized to GAPDH expression and compared with that in parental HEK293 cells (set to 1). The abundance of IGF1R significantly increased in HEKI#25 (1.5-fold), HEKI#36 (2.4-fold), and HEKI#66 (3.3-fold) cells. HEKI#27 cells (1.3-fold) did not show a statistically significant increase (Figure 4A,B). These results indicate that HEKI#25, HEKI#36, and HEKI#66 cells overexpress IGF1R.

The intracellular localization of IGF1R was examined. In both HEK293 cells and HEKI cell lines, IGF1R showed punctate signal patterns at the cell periphery and within the cytoplasm (Figure 4C). Puncta were most pronounced in HEKI#66 cells, consistent with increased IGF1R expression.

**Figure 5:**
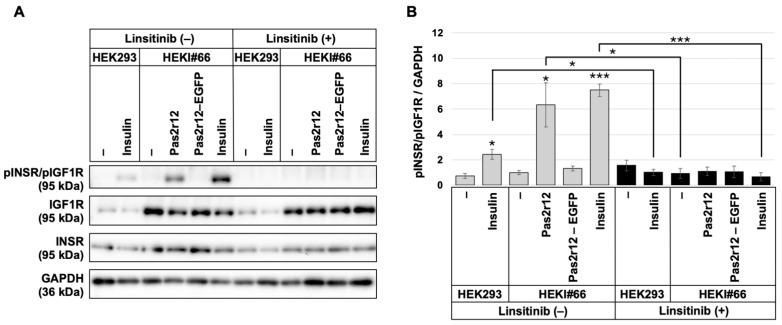
Effects of Pas2r12 or Pas2r12–cargo protein complex stimulation on INSR/IGF1R in IGF1R-overexpressing cells. (**A**) HEKI#66 cells pretreated with DMSO (linsitinib [−]) or linsitinib (linsitinib [+]) were stimulated with Pas2r12, Pas2r12–EGFP, or insulin for 2 min. Level of phosphorylated INSR/IGF1R (pINSR/pIGF1R) was assessed by Western blot. (**B**) Relative levels of pINSR/pIGF1R, normalized to GAPDH, corresponding to the data in panel A. Phosphorylation levels were analyzed using Student’s *t*-test. Each treatment condition was compared with the solvent-treated HEKI#66 control (linsitinib [−]), and, for each treatment, phosphorylation levels in the linsitinib (−) and linsitinib (+) conditions were also compared. Statistically significant differences compared with the solvent-treated control are indicated on the bar graphs, and significant differences between linsitinib (+) and linsitinib (−) treatments are marked by horizontal lines. Error bars indicate the standard error of the mean (SEM). * *p* < 0.05, *** *p* < 0.001. *N* = 3.

We evaluated INSR/IGF1R phosphorylation in HEKI#66 cells following stimulation with Pas2r12 or Pas2r12–EGFP. Compared to the negative control (0.01% DMSO), Pas2r12 induced a significant increase in INSR/IGF1R phosphorylation (6.3-fold; Figure 5A,B); this effect was largely abolished by linsitinib (1.1-fold). Conversely, Pas2r12–EGFP did not significantly alter phosphorylation levels. As a positive control, a 2-min insulin stimulation induced significant INSR/IGF1R phosphorylation (7.5-fold), which was inhibited by linsitinib (0.7-fold).

**Figure 6:**
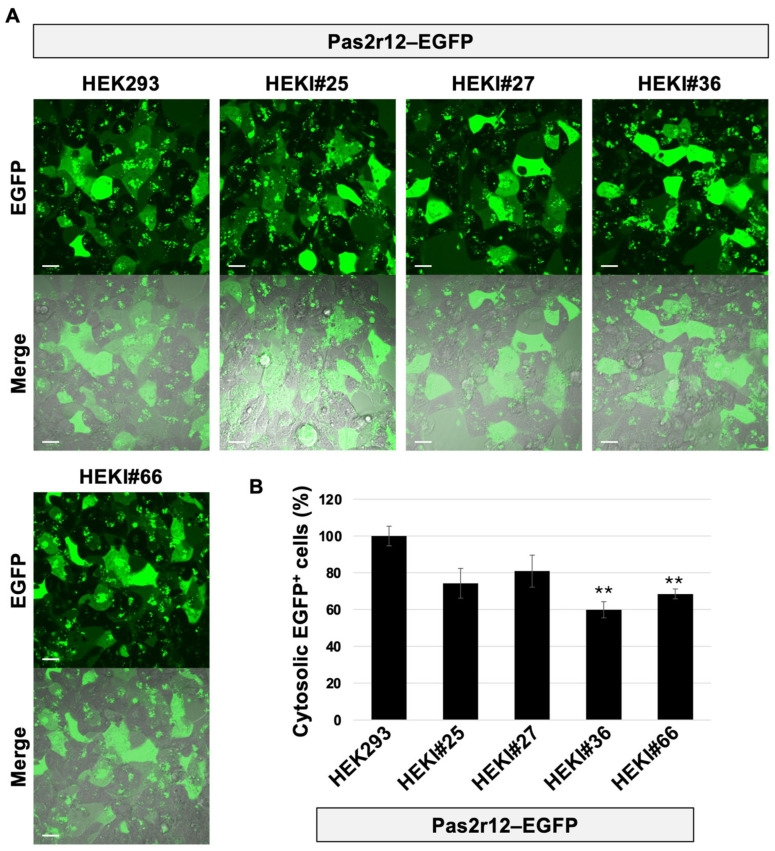
Effect of IGF1R overexpression on the Pas2r12-mediated cytosolic delivery of EGFP. (**A**) Representative confocal images showing the cellular uptake of Pas2r12–EGFP in IGF1R-overexpressing (HEKI) cells. Scale bars represent 20 μm. (**B**) Relative levels of the cytosolic delivery efficiency of EGFP in HEKI cells compared with parental HEK293 cells (set to 100%). Merged images show EGFP fluorescence (green) and differential interference contrast. Statistical comparisons were performed against HEK293 cells using Student’s *t*-test. Error bars indicate the standard error of the mean (SEM). ** *p* < 0.01. *N* = 3.

Finally, Pas2r12-mediated cytosolic delivery of EGFP was evaluated in all cell lines. Using parental HEK293 cells as the reference (100%), delivery efficiency decreased to 74% in HEKI#25, 81% in HEKI#27, 60% in HEKI#36, and 69% in HEKI#66 (Figure 6A,B) cells. Significant reductions were observed in HEKI#36 and HEKI#66 cells, both of which exhibit high IGF1R expression; HEKI#25 and HEKI#27 showed statistically nonsignificant changes. These results indicate that elevated IGF1R expression attenuates Pas2r12-mediated cytosolic delivery of EGFP.

### 2.5. Enhanced Pas2r12-Mediated EGFP Delivery in INSR-Overexpressing Cell Lines

**Figure 7:**
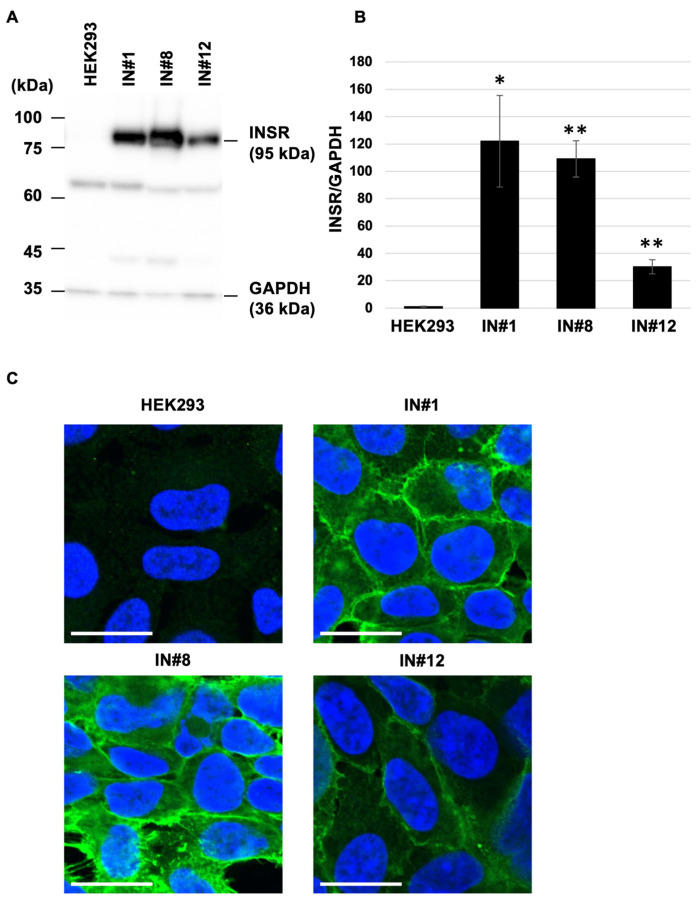
Assessment of INSR overexpression. (**A**) Verification of INSR expression levels in INSR-overexpressing (IN) cells. (**B**) Relative levels of INSR expression normalized to GAPDH, based on the data in panel A. The value for INSR/GAPDH in parental HEK293 cells was set to 1.0 for comparison. Statistical comparisons were performed against HEK293 cells using Student’s *t*-test. Error bars indicate the standard error of the mean (SEM). * *p* < 0.05 and ** *p* < 0.01. *N* = 3. (**C**) Subcellular localization of INSR in IN cells. Green indicates INSR, and blue indicates nuclei. Scale bars represent 20 μm.

To evaluate the effect of INSR overexpression on the Pas2r12-mediated cytosolic delivery of EGFP, INSR levels were analyzed in engineered HEK293-derived cell lines. After normalization to GAPDH and expression compared with parental HEK293 cells (set to 1), INSR expression levels were markedly elevated in all overexpression lines, with increases of ~120-fold in IN#1, 110-fold in IN#8, and 30-fold in IN#12 (Figure 7A,B) cells. These results confirm the substantial overexpression of INSR in all three cell lines.

The intracellular localization of INSR was examined by immunocytochemistry. In parental HEK293 cells, INSR signals were barely detectable, thus preventing reliable localization analysis (Figure 7C). Conversely, strong INSR signals were observed predominantly at the cell periphery in IN#1, IN#8, and IN#12 cells, indicating peripheral enrichment of INSR under overexpression conditions compared with HEK293 cells.

**Figure 8:**
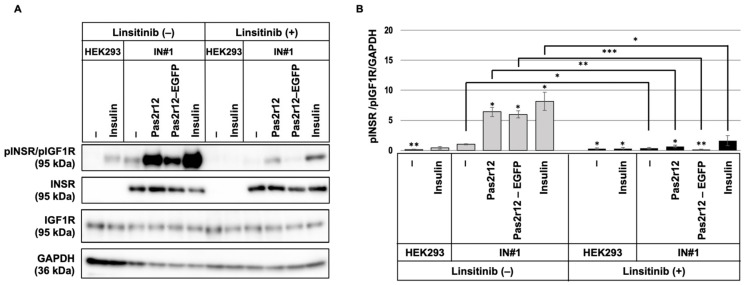
Effects of Pas2r12 or Pas2r12–cargo protein complex stimulation on INSR/IGF1R in INSR-overexpressing cells. (**A**) IN#1 cells pretreated with DMSO (linsitinib [−]) or linsitinib (linsitinib [+]) were stimulated with Pas2r12, Pas2r12–EGFP, or insulin for 2 min. Phosphorylation of INSR/IGF1R (pINSR/pIGF1R) was assessed by Western blot. (**B**) Relative levels of pINSR/pIGF1R were normalized to GAPDH, corresponding to the data in panel A. Phosphorylation levels were analyzed using Student’s *t*-test. Each treatment condition was compared with the solvent-treated IN#1 control (linsitinib [−]), and for each treatment, phosphorylation levels in the linsitinib (−) and linsitinib (+) conditions were also compared. Statistically significant differences compared with the solvent-treated control are indicated on the bar graphs, and significant differences between linsitinib (+) and linsitinib (−) treatments are marked by horizontal lines. Error bars indicate the standard error of the mean (SEM). * *p* < 0.05, ** *p* < 0.01, and *** *p* < 0.001. *N* = 3.

INSR/IGF1R phosphorylation in response to stimulation with Pas2r12–EGFP was analyzed in INSR-overexpressing cells. IN#1 cells were stimulated for 2 min with Pas2r12 or Pas2r12–EGFP. Phosphorylation levels were compared with those of the vehicle control (0.01% DMSO). Stimulation with Pas2r12 or Pas2r12–EGFP induced a substantial increase in INSR/IGF1R phosphorylation (6.4- and 6.0-fold, respectively; Figure 8A,B); these effects were significantly suppressed by linsitinib (0.7- and 0.1-fold, respectively). As a positive control, a 2-min insulin stimulation resulted in a significant increase in INSR/IGF1R phosphorylation (8.2-fold), which was inhibited by linsitinib (1.6-fold). Notably, comparison of basal phosphorylation levels between cell lines showed that vehicle-treated HEK293 cells had basal levels of INSR/IGF1R phosphorylation which was ~0.1-fold of that in IN#1 cells. This finding indicates that INSR overexpression markedly elevates basal receptor activation. Collectively, these findings indicate that in contrast to parental HEK293 cells, Pas2r12–EGFP induces INSR/IGF1R phosphorylation in INSR-overexpressing cells.

**Figure 9:**
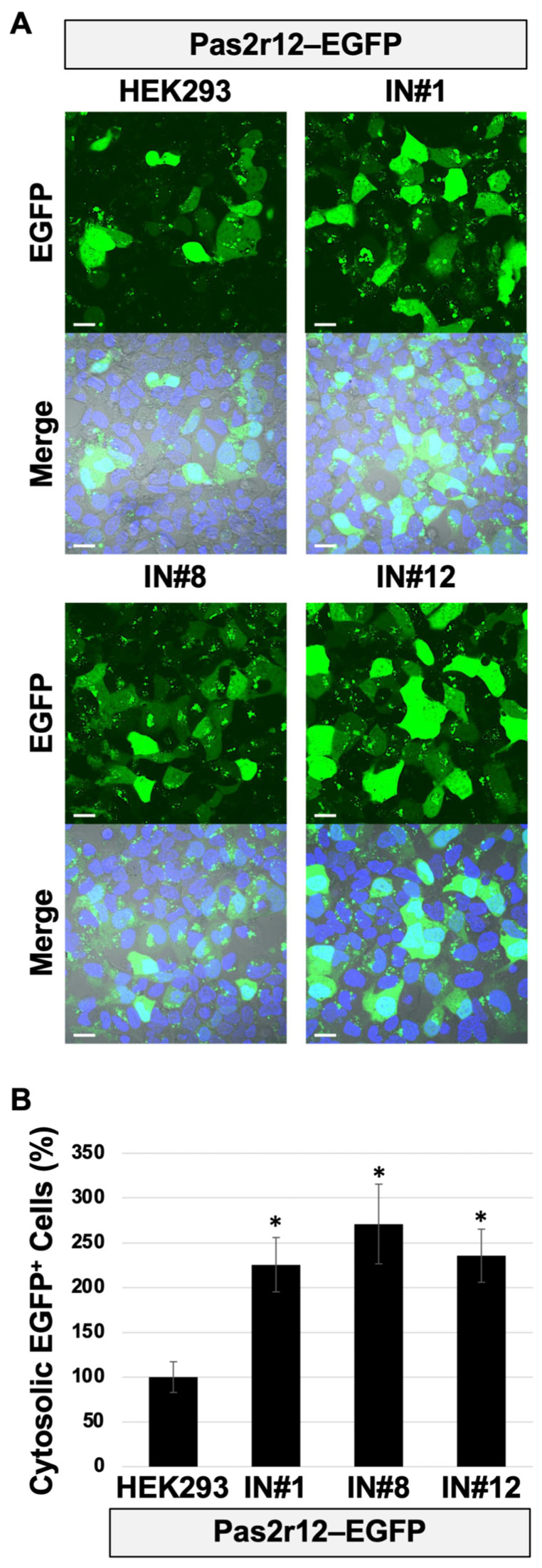
Effect of INSR overexpression on the Pas2r12-mediated cytosolic delivery of EGFP. (**A**) Representative confocal images showing cellular uptake of Pas2r12–EGFP in INSR-overexpressing (IN) cells. Merged images show EGFP fluorescence (green), Hoechst 33342 nuclear staining (blue), and differential interference contrast. Scale bars represent 20 μm. (**B**) Relative levels of the cytosolic delivery efficiency of EGFP in IN cells compared with parental HEK293 cells (set to 100%). Statistical comparisons were performed against HEK293 cells using Student’s *t*-test. Error bars indicate the standard error of the mean (SEM). * *p* < 0.05. *N* = 3.

Finally, Pas2r12-mediated cytosolic delivery of EGFP was evaluated in all cell lines. Compared to parental HEK293 cells (set to 100%), cytosolic delivery efficiency significantly increased to ~230% in IN#1, 270% in IN#8, and 240% in IN#12 (Figure 9A,B) cells. These findings indicate that INSR overexpression substantially enhances the Pas2r12-mediated cytosolic delivery of EGFP.

## 3. Discussion

In this study, we examined the roles of the receptor tyrosine kinases INSR and IGF1R in the Pas2r12-mediated cytosolic delivery of EGFP. In HEK293 cells, stimulation with Pas2r12 transiently increased in INSR/IGF1R phosphorylation; the Pas2r12–EGFP complex did not induce a comparable phosphorylation response. Conversely, in INSR-overexpressing cells, stimulation with Pas2r12–EGFP induced pronounced INSR/IGF1R phosphorylation, suggesting that increased receptor expression enhances ligand–receptor interactions with Pas2r12 and promotes receptor activation. Notably, basal phosphorylation level in parental HEK293 cells was markedly lower (~0.1 compared with IN#1 cells), indicating that INSR overexpression substantially elevates the potential of basal receptor activation. This increase in basal phosphorylation likely reflects enhanced receptor density at the plasma membrane, which can facilitate spontaneous dimerization or increase sensitivity to low levels of endogenous ligands. Alternatively, INSR overexpression may have increased sensitivity to trace levels of endogenous insulin or IGF in the FBS-containing culture medium, resulting in sustained low-level activation. This elevated basal activity likely primes INSR-overexpressing cells for a more robust phosphorylation response upon Pas2r12–EGFP stimulation. Thus, differences in basal receptor activation between cell lines may partially explain why Pas2r12–EGFP-induced phosphorylation was detectable only under INSR-overexpressing conditions. Although phosphorylation was difficult to detect in HEK293 cells, the results from pharmacological inhibition, siRNA-mediated knockdown, and receptor overexpression consistently supported a critical role for INSR in promoting cytosolic delivery.

Interestingly, siRNA-mediated knockdown of INSR or IGF1R led to a reciprocal increase in the protein level of the other receptor. INSR knockdown tended to increase IGF1R expression, whereas IGF1R knockdown enhanced INSR expression. This reciprocal regulation may buffer the effects of single-receptor knockdown on downstream processes, including Pas2r12-dependent cytosolic delivery. Moreover, the markedly different delivery efficiencies observed between siIGF1R_7 (19%) and siIGF1R_8 (65%) may reflect this compensatory mechanism: siIGF1R_8 induced a pronounced increase in INSR expression, which could partially rescue Pas2r12-mediated delivery despite IGF1R knockdown. These findings indicate that compensatory mechanisms should be considered when interpreting our knockdown experiments. Nevertheless, the significant reduction in cytosolic delivery following knockdown of IGF1R or INSR demonstrates that both receptors contribute to, but are not solely responsible for, Pas2r12-mediated protein delivery. Given the potential for heteromeric complex formation between INSR and IGF1R [21,22], it is plausible that INSR–IGF1R heterodimers—or dynamic competition between the two receptors—modulate the internalization efficiency of Pas2r12. In this study, knockdown efficiency was evaluated at the protein level, because the functional consequences of INSR and IGF1R depletion are primarily manifested through changes in receptor protein abundance rather than mRNA levels. Although mRNA levels were not assessed in this study, measuring INSR and IGF1R transcripts in future experiments could provide additional insight into whether the compensatory regulation occurs at the transcriptional level, thereby deepening our understanding of how these receptors coordinate their expression in response to knockdown.

Receptor spatial distribution may influence delivery efficiency. In INSR-overexpressing cells, INSR was predominantly localized at the cell periphery, whereas IGF1R was distributed both at the cell periphery and in the cytoplasm. This difference in localization could contribute to the reduced delivery efficiency observed with IGF1R overexpression. Although increased IGF1R expression was observed in HEKI clones (#25, #36, and #66), it is important to determine whether this increase corresponds to functional activity by assessing IGF1R phosphorylation under the same stimulation conditions. Even with increased IGF1R expression, the receptor may be sequestered within intracellular vesicles, highlighting the value of combining immunocytochemical localization with functional assays using ligands, such as insulin or IGF1.

Notably, the IGF1R-overexpressing lines were generated using a CRISPR-activation system to upregulate endogenous IGF1R, whereas the INSR-overexpressing lines were established through random genomic integration of exogenous INSR. These distinct strategies were adopted because our initial goal was to generate a double-overexpression cell line, and using different systems allowed independent manipulation of each receptor. However, the use of two different overexpression systems may lead to differences in receptor expression patterns, maturation states, or membrane trafficking, potentially influencing Pas2r12 uptake independently of total receptor abundance. Moreover, matched overexpression controls were not included for either receptor system, limiting our ability to interpret the functional consequences of IGF1R or INSR overexpression and to directly compare their contributions. For rigorous comparison, it would be preferable to employ the same overexpression strategy for both receptors and include appropriate system-matched controls. Future studies incorporating these controls and unified expression methods will help more clearly delineate the specific roles of INSR and IGF1R in Pas2r12-mediated cytosolic delivery.

Importantly, ERK1/2 phosphorylation was observed following both Pas2r12 and Pas2r12–EGFP stimulation, independent of INSR/IGF1R inhibition, suggesting that additional receptors or alternative signaling pathways contribute to MAPK activation. Our previous phosphoproteomic analysis showed that Pas2r12 and Pas2r12–EGFP induced the phosphorylation of ephrin-B1 (EFNB1) and lysophosphatidic acid receptor 1 (LPAR1) [16]. EFNB1 is a membrane-anchored ligand that interacts with Eph receptor tyrosine kinases to regulate cell adhesion and cytoskeletal dynamics [29]; LPAR1 is a G protein-coupled receptor involved in various signaling processes, including cell migration and survival [30]. LPAR1 and EFNB1 may therefore mediate ERK1/2 phosphorylation in response to Pas2r12 stimulation.

Beyond INSR and IGF1R, other receptor tyrosine kinases or membrane-associated proteins may independently or cooperatively contribute to Pas2r12–EGFP internalization. Evaluating the phosphorylation status of EGFR and FGFR, which undergo caveolae-dependent internalization, could provide clearer insights into the upstream origin of ERK activation [31,32]. Although EGFR and FGFR phosphorylation was not detected in our previous phosphoproteomic dataset, future analyses using the same approach will be important to determine whether these receptors cooperate with, or function independently of, INSR/IGF1R during Pas2r12–EGFP internalization.

A limitation of this study is the technical challenge of detecting transient and spatially localized phosphorylation events, which may lead to the underestimation of receptor contribution to cytosolic delivery. Phosphorylation of INSR/IGF1R was undetectable in parental HEK293 cells upon Pas2r12–EGFP stimulation, despite functional evidence that INSR and IGF1R contribute to the cytosolic delivery of EGFP. This apparent discrepancy may reflect the highly localized and transient nature of receptor activation, which could occur within specific membrane microdomains, such as caveolae or lipid rafts, and be rapidly reversed during cell lysis or sample handling. Therefore, biochemical detection limits should be considered when correlating receptor phosphorylation with functional uptake. Future studies employing high-sensitivity phosphoproteomics, proximity labeling, or advanced live-cell imaging techniques will be essential to capture transient events and delineate the full receptor network involved in Pas2r12-mediated cytosolic delivery.

Overall, our findings reveal an INSR-dependent mechanism of Pas2r12 uptake and highlight receptor-mediated endocytosis as a key determinant of cytosolic delivery. Although INSR appears to function as a primary mediator, the contribution of IGF1R cannot be excluded, suggesting that heteromeric interactions or cooperative signaling between INSR and IGF1R modulate Pas2r12-mediated uptake. Furthermore, it is necessary to investigate which domain of Pas2r12—Pas2 or r12—is responsible for activating INSR/IGF1R. Additionally, examining other CPPs, such as TAT [33] will be important to determine whether this effect is specific to Pas2r12 or a general property of CPPs. These insights provide a conceptual framework for the rational design of next-generation CPPs with enhanced receptor specificity and optimized intracellular delivery. In particular, strategies that enhance INSR engagement, modulate receptor density, or promote favorable receptor microdomain localization may improve cytosolic delivery performance.

## 4. Materials and Methods

### 4.1. Cell Culture

HEK293 cells (JCRB9068) were obtained from the JCRB Cell Bank. Cells were cultured in Dulbecco’s Modified Eagle Medium (DMEM) supplemented with 10% fetal bovine serum (FBS) at 37 °C in a humidified incubator with 5% CO_2_. Cells were routinely passaged every 5–7 d to maintain optimal growth and viability.

### 4.2. Pas2r12 and EGFP

Pas2r12 (sequence: FFLIGFFLIGrrrrrrrrrrrr-amide; molecular weight: 3046.6), where uppercase letters denote l-amino acids and lowercase letters indicate d-amino acids, was synthesized and purified using Scrum (Tokyo, Japan). The peptide was dissolved in ultrapure water to a final concentration of 1 mM.

His-tagged EGFP was overexpressed in *Escherichia coli* BL21 (DE3) and purified using Ni–NTA agarose chromatography (Qiagen, Hilden, Germany) [34]. Purified EGFP was prepared in phosphate-buffered saline at a final concentration of 0.5 mM.

### 4.3. Reagents

Insulin (Fujifilm Wako, Osaka, Japan) was dissolved in ultrapure water to a final concentration of 50 μM. Linsitinib (Axon Medchem B.V., Groningen, The Netherlands) was dissolved in DMSO to a final concentration of 10 mM.

### 4.4. SDS–PAGE and Western Blotting

HEK293 cells (2 × 10^4^ cells/well) were seeded in 96-well microplates and cultured in 100 μL of DMEM supplemented with 10% FBS until ~80% confluence. The medium was replaced with DMEM containing 2.5% FBS, and the cells were cultured overnight. Based on our previous observations, reducing serum concentration to 2.5% FBS enhanced the cytosolic delivery of cargo proteins by Pas2r12 [8]; therefore, cells precultured under these conditions were used for all experiments.

To prepare the Pas2r12–EGFP complex, Pas2r12 (80 μM) and EGFP (60 μM) were mixed in 25 μL DMEM and incubated for 1 h at 24 °C. The mixture was diluted 1:1 with DMEM to obtain final concentrations of 40 μM Pas2r12 and 30 μM EGFP. Pas2r12 was prepared similarly at 40 μM. The Pas2r12–IgG complex was prepared by mixing Alexa Fluor 488-labeled goat antimouse IgG (H+L) (Abcam, Cambridge, UK; 280 nM) with Pas2r12 (80 μM) in 25 μL DMEM, incubated for 1 h at 24 °C, and diluted 1:1 to final concentrations of 40 μM Pas2r12 and 140 nM IgG. Insulin was diluted in DMEM to a final concentration of 5 μM.

For inhibitor studies, cells were incubated with 1 μM linsitinib in DMEM (2 mL) for 10 min at 37 °C. After pretreatment was complete, the medium was replaced with 50 μL of Pas2r12–EGFP or Pas2r12–IgG complexes diluted 1:1 in DMEM containing 1 μM linsitinib. Vehicle control cells were pretreated with 0.01% (*v*/*v*) DMSO under the same conditions, with complexes diluted in DMEM containing 0.01% DMSO, resulting in a final volume of 50 μL.

Cells were treated for either 2 or 10 min at 37 °C under the following conditions: DMEM (control), Pas2r12, Pas2r12–EGFP, Pas2r12–IgG, or insulin. After treatment was complete, 200 μL of SDS-PAGE sample buffer supplemented with protease inhibitor cocktail (Nacalai Tesque, Kyoto, Japan) and phosphatase inhibitor cocktails II and III (EZBlock; BioVision, Milpitas, CA, USA) was added to each well. Cells were lysed by heating at 95 °C for 10 min.

Lysates were analyzed using 12% SDS–PAGE. Proteins were transferred to polyvinylidene difluoride membranes (GVS, Zola Predosa, Italy) and blocked for 1 h at room temperature using Blocking One or Blocking One P (Nacalai Tesque, Kyoto, Japan). Blocking One P was used to detect phosphorylated proteins. Membranes were incubated with primary monoclonal antibodies against phospho-insulin receptor β (p-IRβ; clone 10C3, sc-81500; Santa Cruz Biotechnology, Dallas, TX, USA), INSR (MAA895Hu21; Cloud-Clone Corp., Houston, TX, USA), phospho-ERK1/2 (sc-7383; Santa Cruz Biotechnology), ERK1/2 (sc-514302; Santa Cruz Biotechnology), and IGF-1 receptor (MAB659Hu22; Cloud-Clone Corp). The antibodies were diluted 1:10,000 in Immuno Shot 1 buffer (Cosmo Bio, Tokyo, Japan). The p-IRβ antibody detects phosphorylation of both IRβ and IGF1R. Anti-GAPDH (clone 3E12; Bioss, Woburn, MA, USA) was used as a loading control at 1:20,000 dilution in antigen–antibody reaction enhancement buffer (Cosmo Bio, Tokyo, Japan).

After incubation with primary antibodies was complete, the membranes were probed with a horseradish peroxidase-conjugated antimouse IgG secondary antibody (Cell Signaling Technology, Danvers, MA, USA) at 1:2500 in Immuno Shot 2 buffer (Cosmo Bio, Tokyo, Japan). Chemiluminescence was developed using ImmunoStar LD detection reagent (Fujifilm Wako, Osaka, Japan) and visualized with a ChemiDoc XRS+ imaging system (Bio-Rad, Hercules, CA, USA). Band intensities were analyzed using Image Lab software (Bio-Rad, Version 6.0.1).

### 4.5. Cytosolic Delivery Assay by Confocal Laser Microscopy

HEK293 cells (4 × 10^5^) suspended in 2 mL of DMEM (Nacalai Tesque, Kyoto, Japan) supplemented with 10% FBS were seeded onto 35-mm glass-bottom culture dishes containing 12-mm glass wells (Iwaki, Tokyo, Japan) precoated with 0.2% gelatin. Cells were incubated at 37 °C in a humidified atmosphere of 5% CO_2_ for 5 d. Then the medium was replaced with DMEM containing 2.5% FBS (2 mL), and the cells were cultured for an 24 h under the same conditions [8].

To evaluate the Pas2r12-mediated cytosolic delivery efficiency of EGFP, cells were subjected to three pretreatment conditions before applying the EGFP–Pas2r12 complex.

In the linsitinib pretreatment condition, the cells were incubated with 1 μM linsitinib in serum-free DMEM (2 mL) for 10 min at 37 °C. Following pretreatment, the medium was removed and replaced with 100 μL of the EGFP–Pas2r12 complex, diluted 1:1 with DMEM containing 1 μM linsitinib. Cells were incubated with the complex for 45 min at 37 °C and washed with 2 mL of DMEM supplemented with 10% FBS.

For the vehicle control condition, cells were pretreated with 0.01% (*v*/*v*) DMSO in serum-free DMEM (2 mL) for 10 min at 37 °C. The EGFP–Pas2r12 complex was diluted 1:1 with DMEM containing 0.01% DMSO (15 μM EGFP and 20 μM Pas2r12: 100 μL) and applied to the cells. Incubation and washing procedures were identical to those used for the linsitinib-treated group.

In the no pretreatment condition (2.5% FBS-DMEM only), cells received no chemical pretreatment before applying the complex. This condition was employed in experiments involving genetic manipulations, such as siRNA-mediated knockdown or IGF1R or INSR overexpression, and served as baseline control for comparison with chemical pretreatment groups.

The EGFP–Pas2r12 complex was prepared by mixing EGFP and Pas2r12 in DMEM at twice the final working concentrations (30 μM EGFP and 40 μM Pas2r12; total volume: 50 μL) and incubating at 24 °C for 1 h to allow complex formation. The resulting complex was diluted 1:1 with plain DMEM (15 μM EGFP and 20 μM Pas2r12: 100 μL) and immediately applied to the cells. Following 45 min of incubation at 37 °C, cells were washed with 2 mL of DMEM containing 10% FBS. For nuclear staining, the cells were incubated for an additional 30 min at 37 °C in DMEM containing 10% FBS supplemented with Hoechst 33342 (500 ng/mL; Thermo Fisher Scientific, Bozeman, MT, USA). The medium was replaced with fresh DMEM (10% FBS) before confocal imaging.

Live-cell imaging was performed using a FluoView FV1200 confocal laser scanning microscope (Olympus, Tokyo, Japan) equipped with a UPlanSApo 60× oil immersion objective lens. Images were acquired at 405 nm (5% laser power) for nuclear staining and 473 nm (5% laser power) for EGFP. For EGFP, the PMT high voltage was fixed at 510 V, with a gain of 1% and offset of 0%. For nuclear staining, the PMT settings were adjusted according to fluorescence intensity, typically with a high voltage of ~680 V, gain of 1%, and offset of 0%. Fluorescence and differential interference contrast images were acquired and overlaid.

Cells exhibiting cytosolic EGFP fluorescence were identified by visual inspection. The proportion of EGFP-positive cells was calculated as a percentage of the total cell population, determined by counting nuclei when Hoechst 33342 was used or by differential interference contrast images when nuclear staining was not used. For each experimental condition, 3–5 fields per sample were analyzed. Experiments were independently repeated at least three times, and the results are presented as mean ± standard error of the mean.

### 4.6. siRNA Transfection

HEK293 cells were seeded in 35 mm glass-bottom dishes at 2 mL in DMEM supplemented with 10% FBS and incubated for 72 h until ~70% confluence. siRNAs targeting *IGF1R* (Hs_IGF1R_7: TCGAAGAATCGCATCATCATA, Hs_IGF1R_8: CTGGACTCAGTACGCCGTTTA), *INSR* (Hs_INSR_2: TCGAACGATGTTGGACTCATA, Hs_INSR_3: CAACGGGAGTCTGATCATCAA), and a negative control siRNA (siNC: AATTCTCCGAACGTGTCACGT) were obtained from QIAGEN (Venlo, The Netherlands).

Transfection was performed using Lipofectamine RNAiMAX (Thermo Fisher Scientific), following the manufacturer’s protocols. Briefly, 25 pmol of siRNA was diluted in 125 µL Opti-MEM, and 7.5 µL of RNAiMAX was diluted in 125 µL Opti-MEM. The two solutions were combined, incubated at room temperature for 5 min, and added dropwise to the cells. After 48 h, the medium was replaced with DMEM containing 2.5% FBS (2 mL), and cells were incubated for an 24 h before downstream analyses.

### 4.7. Establishment of an IGF1R-Overexpressing Stable Cell Line

HEK293 cells (4 × 10^5^) were seeded into 6-well plates containing 2 mL of DMEM supplemented with 10% FBS and cultured until 70–90% confluence. Transfection was performed using Lipofectamine 3000 with 5 µg of the IGF1R CRISPR Activation Plasmid (sc-400084-ACT; Santa Cruz Biotechnology). After 48 h, the medium was replaced with DMEM containing 10% FBS and a combination of puromycin (0.8 µg/mL), blasticidin (20 µg/mL), and hygromycin B (300 µg/mL). After 2 d of selection, cells were trypsinized and plated at appropriate dilutions onto 60-mm dishes to facilitate colony formation. After 10 d of incubation at 37 °C, individual antibiotic-resistant colonies were manually picked and transferred to fresh selection medium. Clones that maintained antibiotic resistance after three passages were evaluated for IGF1R expression using Western blot.

### 4.8. Generation of an INSR-Expressing Stable Cell Line

To generate INSR-overexpressing HEK293 cells, the plasmid pRP[Exp]-Neo-CAG > hINSR (VectorBuilder, Chicago, IL, USA; 20 µg) was linearized using *Sca* I. HEK293 cells (2 × 10^5^ cells/mL, 2 mL per well) were seeded into 6-well plates and cultured until 70–90% confluence. Transfection was performed using Lipofectamine 3000. Briefly, 5 µL of Lipofectamine 3000 was mixed with 125 µL of Opti-MEM. Separately, 2.5 µg of linearized plasmid DNA was combined with 10 µL of P3000 reagent in 125 µL of Opti-MEM. The two solutions were mixed, incubated at room temperature for 10 min, and applied to the cells. After 48 h, the culture medium was replaced with DMEM supplemented with 10% FBS and 100 µg/mL G418 for selection. Cells were diluted 1:80, cultured for 48 h, plated on 60-mm dishes, and incubated for 10 d to allow colony formation. Individual G418-resistant colonies were manually picked and transferred to 96-well plates containing selection medium. Cells were passaged four times under continuous G418 selection. INSR overexpression was confirmed using Western blot.

### 4.9. Immunohistochemical Staining

HEK293 cells (2 × 10^5^ cells) were seeded onto Millicell EZ 8-well glass slides and cultured in 0.5 mL of DMEM supplemented with 10% FBS until they were 80% confluent. The medium was replaced with DMEM containing 2.5% FBS. The cells were incubated for 24 h; fixed with 4% paraformaldehyde; permeabilized with phosphate-buffered saline containing 0.2% Triton X-100; blocked with Blocking One at room temperature; incubated overnight at 4 °C with primary antibodies against IGF1R (3B7, sc-462; Santa Cruz Biotechnology) or INSR (CT3, sc-57342; Santa Cruz Biotechnology) diluted 1:200 in Immuno Shot 1 solution; washed; and incubated at room temperature for 45 min with secondary antibody Alexa Fluor 488-labeled goat antimouse IgG and Hoechst 33342 diluted 1:500 in Immuno Shot 2 solution. Cells were mounted using Dako Fluorescence Mounting Medium (Agilent, Santa Clara, CA, USA) and imaged using a confocal laser scanning microscope.

### 4.10. Statistical Analysis

Statistical significance was assessed using Student’s *t*-test, with results considered significant at * *p* < 0.05, ** *p* < 0.01, and *** *p* < 0.001.

## 5. Conclusions

Pas2r12 mediated the cytosolic delivery of EGFP in HEK293 cells primarily through INSR-dependent uptake. INSR overexpression increased receptor phosphorylation and cytosolic delivery; IGF1R contributed in a secondary or cooperative manner, potentially through heteromeric INSR–IGF1R complexes or shared downstream trafficking pathways. Knockdown studies confirmed that both receptors are important, although neither alone fully accounted for Pas2r12-mediated uptake.

In addition, Pas2r12 and Pas2r12–EGFP induce ERK1/2 phosphorylation, suggesting that additional receptors or signaling pathways may participate in cargo internalization. Future studies examining the functional domains of Pas2r12 involved in receptor activation and comparisons with other CPPs will help determine whether these effects are specific to Pas2r12.

These findings provide a framework for the design of next-generation CPPs with enhanced receptor specificity, optimized cytosolic delivery, and potential applications for selective intracellular targeting.

## Data Availability

The original contributions presented in this study are included in the article/supplementary material. Further inquiries can be directed to the corresponding author.

## Supplementary Materials

The following supporting information can be downloaded at: https://www.mdpi.com/article/10.3390/ph18121885/s1, Figure S1: Cytosolic delivery of EGFP with or without Pas2r12 in HEK293 cells.

## Abbreviations

The following abbreviations are used in this manuscript:

CPPs	Cell-penetrating peptides
DIC	Differential interference contrast
DMEM	Dulbecco’s Modified Eagle Medium
EGFP	Enhanced green fluorescent protein
FBS	Fetal bovine serum
ERK1/2	Extracellular signal–regulated kinase 1/2
INSR	Insulin receptor
IGF1R	Insulin-like growth factor 1 receptor
MAPK	Mitogen-activated protein kinase
PBS	Phosphate-buffered saline
RTKs	Receptor tyrosine kinases
