# Supplementary figures and images for: Role of the Insulin Receptor in Mediating Cytosolic Delivery of Proteins by a Modified Cell-Penetrating Peptide
Source: Pharmaceuticals (Basel). 2025 Dec 12;18(12):1885. doi: 10.3390/ph18121885 (PMC12736253; doi:10.3390/ph18121885)

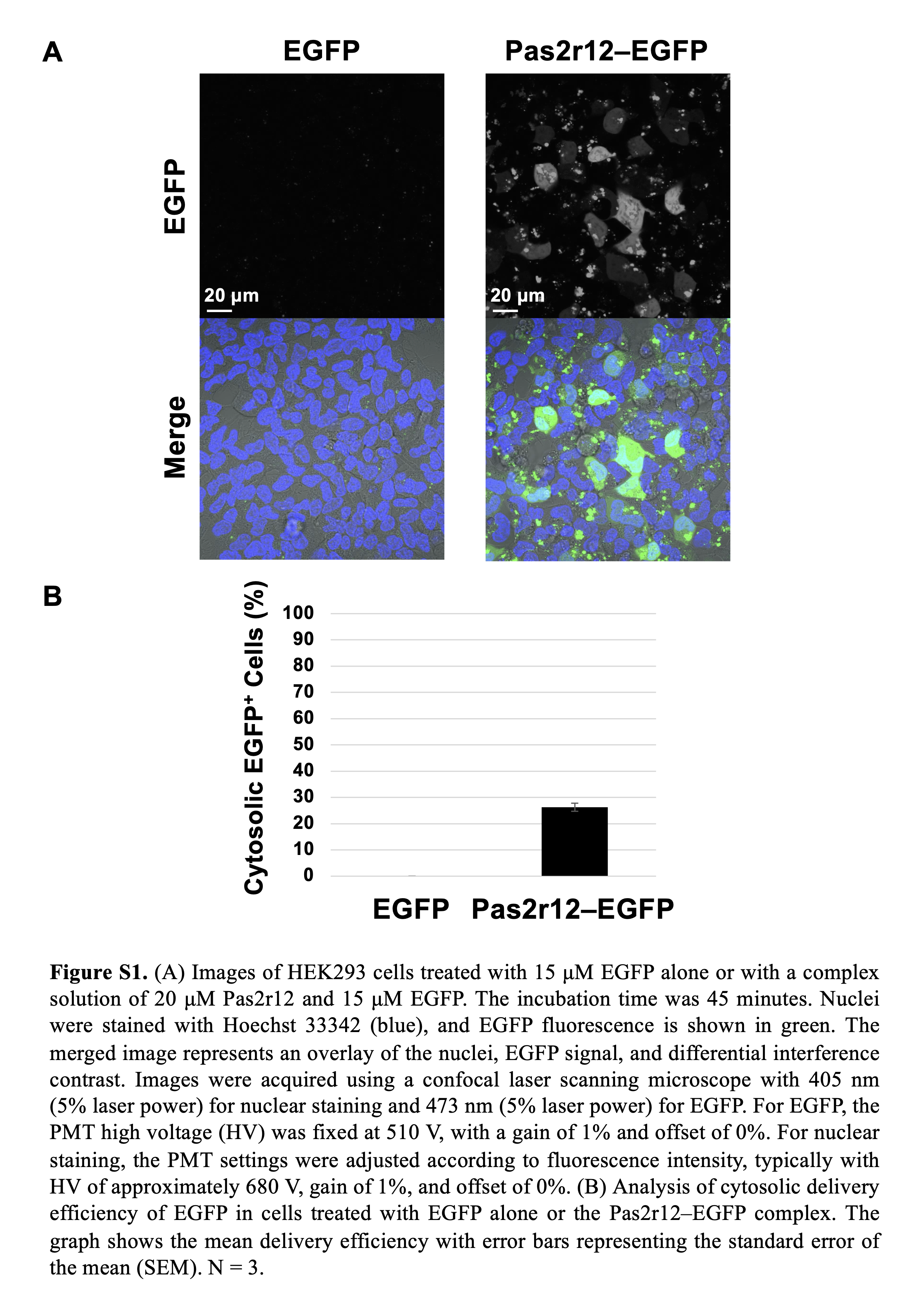

Supplement: Supplementary file 1 [file pharmaceuticals-18-01885-s001.zip › pharmaceuticals-4020832-supplementary.tiff]
